# Temporal Patterns in Bacterioplankton Community Composition in Three Reservoirs of Similar Trophic Status in Shenzhen, China

**DOI:** 10.3390/ijerph13060599

**Published:** 2016-06-16

**Authors:** Jiancheng Li, Cheng Chen, Jun Lu, Anping Lei, Zhangli Hu

**Affiliations:** 1 Shenzhen Key Laboratory of Marine Bioresource and Eco-Environmental Science, Guangdong Engineering Research Center for Marine Algal Biotechnology, College of Life Science, Shenzhen University, Shenzhen 518060, China; lijc007@szu.edu.cn (J.Li); chencheng_sz@126.com (C.C.); jun.lu@aut.ac.nz (J.Lu); 2 School of Science, Faculty of Health and Environmental Sciences, Auckland University of Technology, Auckland 1142, New Zealand

**Keywords:** bacterioplankton community composition (BCC), PCR-DGGE, reservoir, trophic status

## Abstract

The bacterioplankton community composition’s (BCC) spatial and temporal variation patterns in three reservoirs (Shiyan, Xikeng, and LuoTian Reservoir) of similar trophic status in Bao’an District, Shenzhen (China), were investigated using PCR amplification of the 16S rDNA gene and the denaturing gradient gel electrophoresis (DGGE) techniques. Water samples were collected monthly in each reservoir during 12 consecutive months. Distinct differences were detected in band number, pattern, and density of DGGE at different sampling sites and time points. Analysis of the DGGE fingerprints showed that changes in the bacterial community structure mainly varied with seasons, and the patterns of change indicated that seasonal forces might have a more significant impact on the BCC than eutrophic status in the reservoirs, despite the similar Shannon-Weiner index among the three reservoirs. The sequences obtained from excised bands were affiliated with *Cyanobacteria*, *Firmicutes*, *Bacteriodetes*, *Acidobacteria*, *Actinobacteria*, *Planctomycetes*, and *Proteobacteria*.

## 1. Introduction

Bacterioplankton play an important role in the food web of both lotic and lentic water bodies, serving as a regulator of oxygen, carbon, and nutrient dynamics [1]. Since bacterioplankton are sensitive to both hydrologic and water quality changes and respond rapidly to these changes, they are considered to be a good indicator of water quality and have attracted considerable attention in recent years in a variety of water habitats [2,3,4,5]. Most of the studies have been focused on temperate and tropical regions [4,6,7,8,9], and information on sub-tropical systems is scarce.

Bacterial communities can respond rapidly to seasonal changes, such as algal abundance, grazing pressure, and concentrations of total nitrogen (TN) and total phosphate (TP) [10,11,12]. In addition, the activity of the bacterial community can be strongly dependent on temperature, light levels, and gross primary productivity that exhibit seasonal fluctuations. Thus, patterns of change in bacterial community composition (BCC) should reflect the effects of lake-specific characteristics, as well as the effects of parameters that fluctuate seasonally. To obtain insights into the dynamics of bacterial populations in lake or reservoir ecosystems, it is important to explore temporal and spatial variations of BCC. Although a lot of multi-lake or multi-reservoir studies have shown variation in BCC among different lakes of different trophic status, little is known about such variations in freshwater reservoirs of similar trophic status [5,6,7].

The analysis of BCC by classical taxonomic identification has historically been a difficult task because of their small size and lack of distinguishing features. Moreover, culturable strains with current technology are not necessarily representative of either the composition or diversity of natural bacterial communities, since most of the bacterial species (>90%) are at a Viable But Not Culturable (VBNC) state [13,14,15]. In the past two decades, 16S rDNA-based community fingerprinting techniques, such as denaturing gradient gel electrophoresis (DGGE) and terminal restriction fragment length polymorphism (T-RFLP), have been developed which can generate unique bacterial community signals from bulk DNA samples. The successful application of this molecular method has provided the means of obtaining information about the BCC’s dynamics in the natural environment [16,17,18]. Although the DGGE banding patterns may not reflect the community fully, PCR-DGGE was a commonly used molecular method to investigate the spatial-temporal dynamics of BCC in natural environments [4,19,20].

In the present study, the genetic diversity of the bacterioplankton in three shallow reservoirs of similar trophic status in Shenzhen (China) were investigated using PCR-DGGE fingerprinting. The temporal variations patterns of BCC were derived from a year-round monthly sampling. This study aims to (1) compare the spatial and temporal variations of BCC in three reservoirs of similar trophic status; (2) examine whether BCCs are similar at those three reservoirs with similar trophic status.

## 2. Materials and Methods

### 2.1. Study Sites

**Figure 1:**
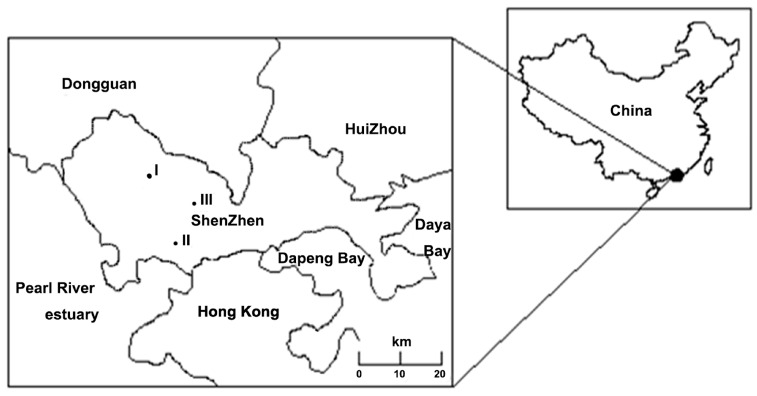
Location of the study area (I: Shi-yan Reservoir; II: Xi-keng Reservoir; III: Luo-tian Reservoir), and Shenzhen region (south of Guangdong Province).

Three main reservoirs (Shiyan, Xikeng, and Luotian Reservoir) in Bao’an District (22°24′N, 114°08′E), Shenzhen (Guangdong Province, China), were selected in the present study (Figure 1). Their important tributary is the Dongjiang River, and they contribute to the Shenzhen region’s sources of drinking water. Carlson’s Trophic State Index (TSI) [21] is one of the most commonly used trophic indices, and it is the trophic index used by the United States Environmental Protection Agency. The TSI values of the three reservoirs were higher than 53 (varied from 53.99 to 60.17), indicating that these reservoirs were in the eutrophication status. The analyzed reservoirs are small and shallow reservoirs, and a summary of the physico-chemical characteristics is given in Table 1.

### 2.2. Sample Collection

Sampling for the bacterial community was performed at monthly intervals. Since the selected reservoirs were shallow, samples were collected from approximately 50 cm below the surface by using a five-liter organic glass water sampler, which was cleaned by rinsing with water from the specific location and sealed after overflow of sampling water to avoid any air bubbles. Reservoirs were sampled within a 2–3 h duration (9:00–12:00 A.M.) on the same day. Concurrent with bacterioplankton sampling, the water temperature and transparency were determined *in situ*. Samples were stored in 2-L pre-sterilized polypropylene bottles, transported to the laboratory and stored in the dark at 4 °C until analysis. In the lab, 1000–2000 mL water samples were filtered by 0.2-μm polycarbonate filters (diameter 50 mm, Millipore, Billerica, MA, USA) and the polycarbonate filters were then stored at −20 °C until DNA extraction. Environmental variables were measured for water samples, including total phosphorus, total nitrogen, Chlorophyll *a*, Dissolved Organic Carbon (DOC), Permanganate Index, and the modified trophic state index (TSI_M_), using standard methods stated in the literature [22].

### 2.3. Total Bacterioplankton Counts

The total bacterial abundance in each sample was determined by direct fluorescence microscopic counts by using Olympus BX51, and the Live/Dead Bacterial Viability Kit (Invitrogen, Waltham, MA, USA) according to the manufacturer’s protocol. With the excitation of 488 nm and emission of 510 nm under the observation by Olympus BX51 (Olympus, Tokyo, Japan), living bacteria exhibit green fluorescence. The 200 μL water samples yielded 20–100 stained cells in a counting field of a standard cytometer, and a minimum of 400 cells were counted.

### 2.4. Total Microbial DNA Extraction and 16s rDNA-V3 PCR Amplification

The polycarbonate filters with bacterioplankton were cut into small pieces with a sterile scalpel, and total DNA was extracted according to the protocol of the wizard genomic DNA purification kit (Promega, Fitchburg, WI, USA). The purity of DNA was assessed by electrophoresis in 1% agarose gels, and DNA was stored at −20 °C before PCR amplification. 

16S rDNA amplification was carried out in a touch-down PCR. In the first-round PCR, the 50-μL PCR mixture contained 0.5 μL template DNA solution (approximately 200 ng DNA), 0.5 μL each primer bact341f (5′-CCTACGGGAGGCAGCAG-3′) and 518r (5′-ATTACCGCGGCTGCTGG-3′) [23], which were designed to amplify 16S rDNA-V3 segments from all members of the bacteria, 10 μL (0.5 units) Premix Taq DNA polymerase (Takara, Otsu, Japan), and 8.5 μL ddH_2_O. The reactions were carried out as follows: 5-min initial incubation at 94 °C, followed by 20 cycles of 30 s denaturation at 94 °C, 30 s annealing at 65 °C, and 30 s extension at 72 °C.

Second-round PCR was performed with primers 518r and DGGE-341f (5′-CGCCCGCCGCGCGCGGCGGGCGGGGCGGGGGCACGGGGGGCCTACGGGAGGCAGCAG-3′) [24,25]. DGGE-341f was designed by attaching 40 bp GC-clamps to 341f in order to increase the separation of DNA bands in the DGGE gel. The PCR mixture contained 1.0 μL DNA sample obtained from the first-round PCR (approximately 80 ng DNA), 0.5 μL each primer, 25 μL (1.25 units) Premix Taq DNA polymerase, and 23.0 μL ddH_2_O. The reactions were carried out as follows: 5-min initial incubation at 94 °C, followed by 20 cycles of 30 s denaturation at 94 °C, 30 s annealing at 65 °C (0.5 °C decrease for every cycle), and 30 s extension at 72 °C; five additional cycles were carried out at an annealing temperature of 55 °C, and followed by incubation for 10 min at 72 °C. The yield and quality of PCR products was determined by electrophoresis on 1.2% agarose gels and stained with ethidium bromide.

### 2.5. DGGE

DGGE was performed using the DCode™ system (Bio-Rad, Hercules, CA, USA). PCR samples were loaded onto 10% polyacrylamide (acrylamide/bis, 37.5:1) gels in 0.5 × TAE (20 mM Tris acetate, pH 7.4, 10 mM sodium acetate, 0.5 mM Na2–EDTA). The denaturing gradient contained 30%–70% denaturant (100% denaturant is a mixture to 7 M urea and 40% (vol/vol) formamide) [26]. Electrophoresis was performed at conditions of 60 °C, 50 V with a run time of 30 min, and then 75 V with a run time of 14 h. After electrophoresis, the gels were stained with 100 mL 3X GelRed and shaken on a rotary shaker at 120 rpm for 2 h, and then gels were placed on a UV trans-illuminator and photographed with Bio-Rad Gel Doc 2000 equipment.

The DGGE profiles were analyzed using Quantity One software 4.6.2 (Bio-Rad). First, the DGGE banding patterns were converted to a binary matrix using presence-absence data, (*i.e.*, 1 represents presence and 0 represents absence), and then a pairwise similarity of the banding patterns of the different samples in each reservoir was calculated with the Dice coefficient. Using these pairwise similarity values, an unweighted pair group with mathematical averages (UPGMA) cluster analysis was performed to obtain the sequence similarity matrices. In addition, the peak area from the DGGE footprint was estimated by Gel-Pro Analyzer software, and then the Shannon index-H, a parameter for the bacterial community diversity, was calculated according to the method described previously [27].

### 2.6. Sequencing and Phylogenetic Analysis of Excised DGGE Bands

Individual bands were cut from the DGGE gel using razor blades, placed in 100 μL sterile distilled water, and incubated overnight at 37 °C. One microliter of the eluted band was re-amplified with touchdown PCR as described in Section 2.4, and the re-amplified bands were reanalyzed by DGGE to ensure purity before sequencing. 

The sequencing reactions were performed by Beijing Genomics Institute-Shenzhen (BGI-SZ). The PCR products of the excised bands were sequenced directly, the primer set for sequence analysis, T7-bact341f (5′-TAATACGACTCACTATAGGGCCTACGGGAGGCAGCAG-3′) and M13R-518r (5′-CAGGAAACAGCTATGACCATTACCGCGGCTGCTGG-3′), were designed based on bact341f and 518r, respectively. The PCR was performed using the same program as 16S rDNA-V3 amplification.

The sequences were compared to sequences stored in GeneBank using the BLAST algorithm. Subsequently, the sequences were imported into the DNAStar software program, aligned using the MegAlign tool, and a phylogenetic tree was constructed.

## 3. Results

### 3.1. Seasonal Fluctuations in Trophic State Index (TSI)

**Figure 2:**
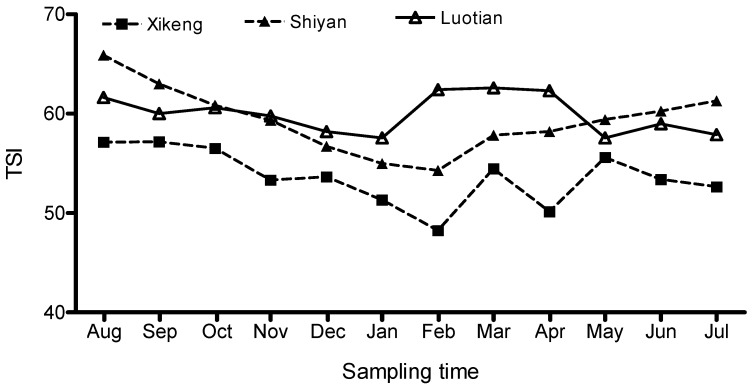
Trophic state index (TSI) value in the three reservoirs from August to July.

Spatial and seasonal fluctuations of the TSI of the three reservoirs are shown in Figure 2. The TSI seasonal variation of three reservoirs showed a similar trend, decreasing from August, reaching a minimum around February, and then increasing slowly again. The TSI in the hot wet months of late summer and early autumn (May to October) were generally higher than that in the mild dry months of winter and spring (November to April). However, the TSI in Luo-tian Reservoir was a bit higher than the other two in late winter and early spring (February to April). Spatially, the TSI value was similar among the three reservoirs, although it was slightly lower in Xi-keng than that in the other two reservoirs. These results indicated that trophic status was similar among the three reservoirs, and it was slightly less in the Xi-keng Reservoir than the other two.

### 3.2. Seasonal Fluctuations in Total Bacterioplankton Abundance

**Figure 3:**
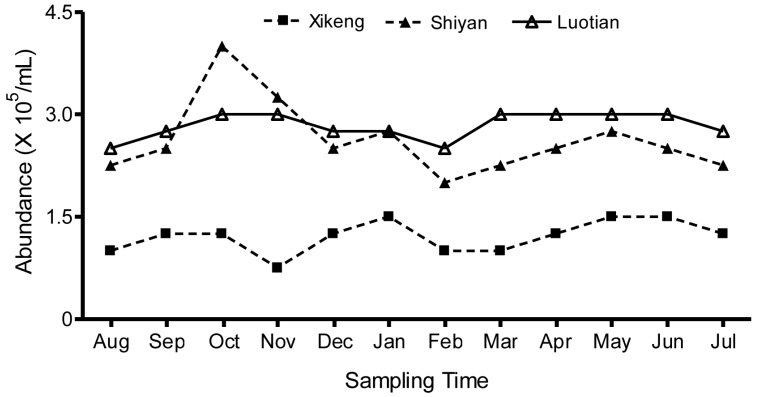
Bacterioplankton abundance in three reservoirs.

The total bacterioplankton abundance ranged from 7.5 × 10^4^ to 4 × 10^5^ cells/mL in the three reservoirs during the sampling period. Spatially, monthly average bacterioplankton abundances of 2.8 × 10^5^, 2.6 × 10^5^ and 2.1 × 10^4^ cells/mL were observed in Luo-tian, Shi-yan, and Xi-keng Reservoir, respectively; and the abundance declined in the order from the higher to the slightly lower trophic status reservoir. Seasonally, the bacterioplankton were more abundant in October and May, while less abundance was observed in February (Figure 3). Among the three reservoirs, the range of abundance of Shi-yan displayed a dramatic shift with the seasons, and the other two were smoother.

### 3.3. Bacterioplankton Community Composition

#### 3.3.1. DGGE Fingerprint Patterns

**Figure 4:**
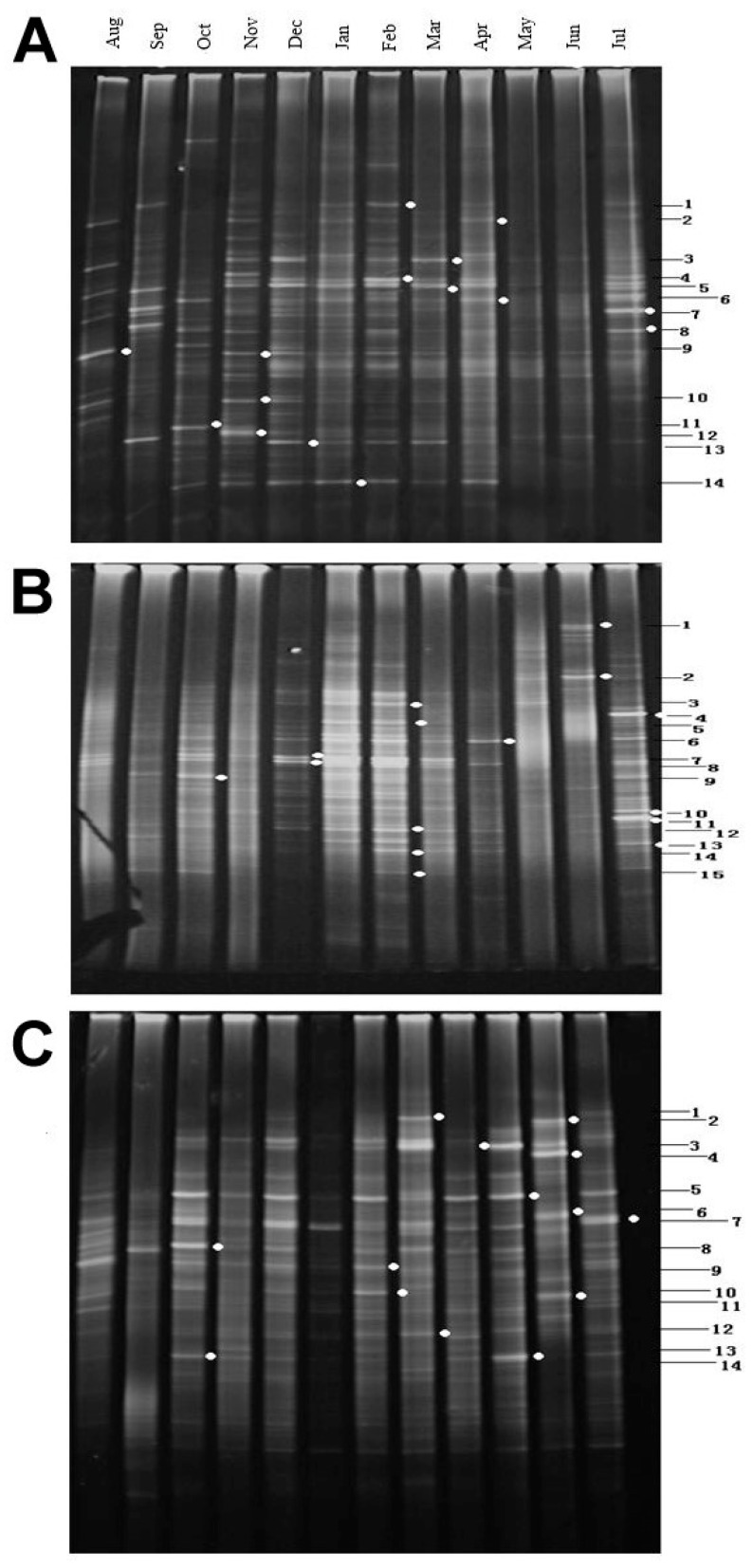
Denaturing gradient gel electrophoresis (DGGE) fingerprints of the samples from three reservoirs. Dots show the bands that were analyzed by sequencing. (**A**) Xi-keng Reservoir; (**B**) Shi-yan Reservoir; (**C**) Luo-tian Reservoir.

The DGGE fingerprint patterns obtained from the water samples are shown in Figure 4. Generally, the bands were distinct and dispersed across the entire gel gradient. In each reservoir, one or two DGGE bands were found in all the samples, though the intensity of bands differed with months, and most bands were present in one or several months. The DGGE patterns and the dominant bands were different, depending on the reservoirs and sampling time, indicating that bacterioplankton community composition was different among reservoirs and sampling time. In general, the variation in DGGE profiles was higher between samples from different sampling times at the same sampling site than that between samples obtained from the same sampling time in different reservoirs.

#### 3.3.2. Bacterioplankton Richness and Shannon-Weiner Index

**Figure 5:**
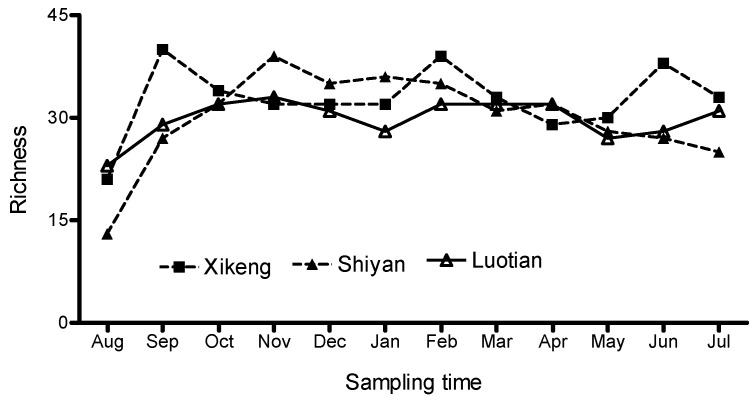
Bacterioplankton richness in the three reservoirs. The number of DGGE bands per gel is regarded as a representation of richness.

Figure 5 shows the bacterioplankton richness, which was defined as the number of DGGE bands. Spatially, although the number of bands for the individual samples varied from 13 to 40, the monthly average bacterioplankton richness did not vary greatly among reservoirs (30–33), and the richness was slightly higher in the Xi-keng Reservoir, which has slightly less trophic status than the other two reservoirs. Seasonally, the bacterioplankton richness remained relatively stable from late autumn to early spring, while it fluctuated significantly in other times, especially from August to September, with the lowest richness observed in August and then a sharp increase.

**Figure 6:**
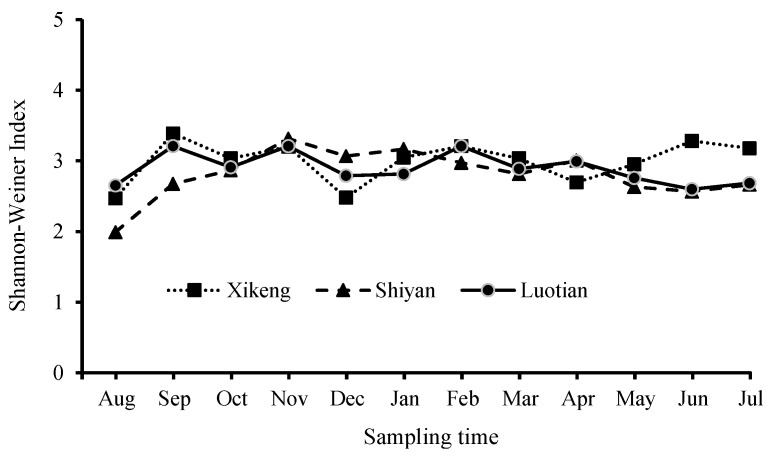
Shannon-Weiner index of three reservoirs.

Similar to the bacterioplankton richness, the Shannon-Weiner richness index did not vary greatly among the three reservoirs (2.8–3.0) (Figure 6). Seasonally, the index remained relatively stable throughout the year, although it fluctuated in June to September of late summer and early autumn, with the lowest (2.02) and the highest (3.37) index observed in August and September/November, respectively.

#### 3.3.3. Cluster Analysis

**Figure 7:**
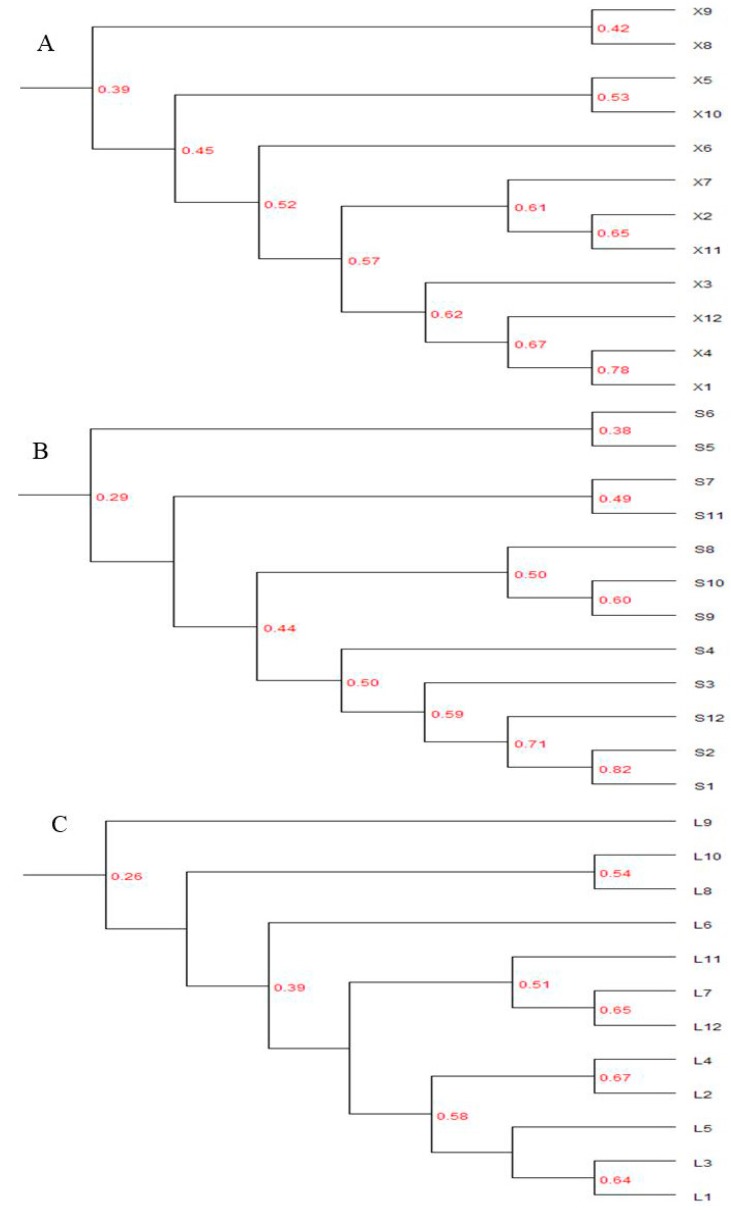
Dendrogram obtained by unweighted pair group with mathematical averages (UPGMA) clustering of DGGE patterns from three reservoirs. Similarity is expressed as the Dice correlation coefficient. (**A**) Xi-keng (X) Reservoir; (**B**) Shi-yan (S) Reservoir; (**C**) Luo-tian (L) Reservoir.

Cluster analysis by UPGMA in each reservoir showed that there was low similarity of BCC among the samples from different months, and the BCCs were grouped into four clusters (Figure 7). In the Xi-keng Reservoir, BCCs in August and September formed one cluster, BCCs in May and October were grouped into another cluster, BCCs in June alone formed one cluster, and those in the other months tended to cluster (Figure 7A). In the Shi-yan Reservoir, BCCs in May and June formed one cluster, BCCs in July and November were grouped into one cluster, BCCs in August, September, and October formed another cluster, and those in the other months tended to cluster (Figure 7B). In the Luo-tian Reservoir, BCCs in September and June formed two clusters, BCCs in August and October were grouped into one cluster, and those in the others formed another cluster (Figure 7C). 

Despite the among-lake differences observed in this study, variation in the bacterial communities as assessed by DGGE fingerprints showed some general patterns. BCCs in December, January, February, March, and April were in the same cluster in all reservoirs; while in late spring to autumn, two to three months, or even one month alone, of BCCs formed one cluster. Results of the UPGMA cluster analysis suggested that the bacterioplankton communities in winter and early spring appeared to be much more stable than those in late summer and early autumn periods.

#### 3.3.4. Sequencing and Phylogenetic Analysis

The major DNA bands in the DGGE gel (dots in Figure 4) were selected for PCR re-amplification and then sequence analysis. These sequences were submitted to GeneBank (KX348149–KX348153). Based on the BLAST analysis, only one cultured bacterium was identified in each reservoir, and the majority of the bacteria were uncultured. A great deal of overlaps in the composition of bacterial communities among different lakes were found, although their accession number in GeneBank might be different. 

**Figure 8:**
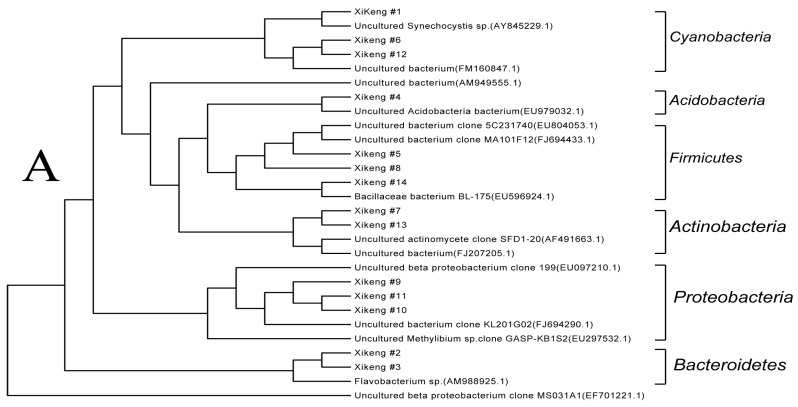

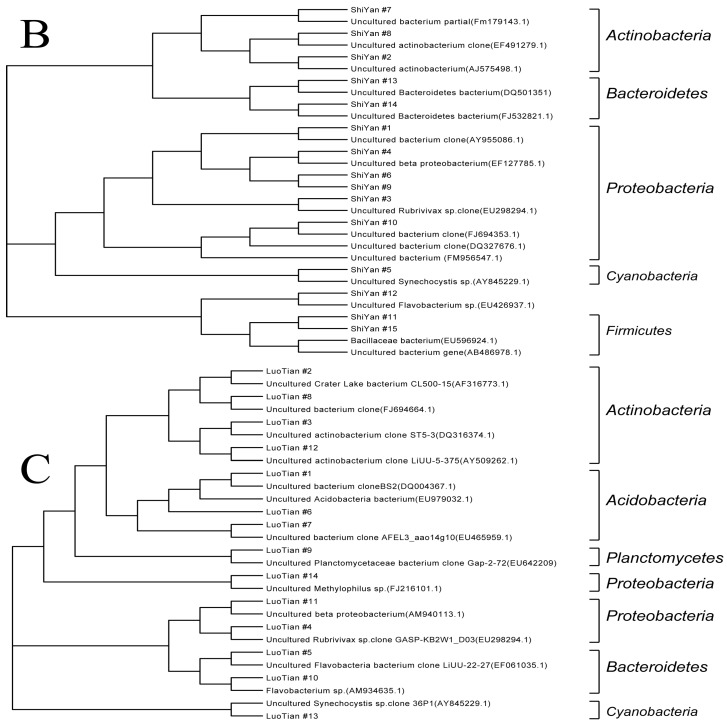
Phylogentic tree based on the excised DGGE band of three reservoirs. (**A**) Xi-keng Reservoir; (**B**) Shi-yan Reservoir; (**C**) Luo-tian Reservoir.

Figure 8 shows the phylogenetic relationship of the 16S rDNA-V3 sequences representing the respective excised DGGE bands in the three reservoirs. The bacterial sequences were grouped into five to seven clusters in each reservoir. The clusters are *Planctomycetes*, *Cyanobacteria*, *Bacteroidetes*, *Firmicutes*, *Proteobacteria*, *Acidobacteria*, and *Actinobacteria*. Obviously, the BCC was different among the three reservoirs (Table 2). *Proteobacteria*, *Actinobacteria*, *Bacteroidetes*, and *Cyanobacteria* were found in all the three reservoirs, and they constituted the majority of the bacterial community. *Proteobacteria, Actinobacteria*, and *Bacteroidetes* were most dominant in the Shi-yan Reservoir, and account for 40%, 20%, and 20% of the BCC, respectively. While for the Luo-tian Reservoir, which has almost the same trophic status as Shi-yan Reservoir, *Actinobacteria* and *Acidobacteria* were most abundant, and accounted for 28.6% and 21.4% of the BCC, respectively. *Firmicutes*, *Cyanobacteria*, and *Proteobacteria* were most dominant in the slightly lower trophic Xi-keng Reservoir, and accounted for 28.6%, 21.4%, and 21.4% of the BCC, respectively.

## 4. Discussion

### 4.1. Factors Affecting Patterns of BCC

Bacterial communities can respond rapidly to changes that occur on seasonal scales, such as grazing pressure, viral count, algal abundance, and nutrient concentrations. Thus, seasonal forces may be the overriding factors of the BCC irrespective of the dynamics of the individual reservoir water system components [7,12]. The patterns of bacterial community change in the present study indicate that seasonal forces are more important in influencing the behavior of the bacterial communities in each reservoir. All three reservoirs have relatively stable BCCs in winter and early spring, but in the late summer and early autumn the changes are dramatic. The BCCs are quite different among the three reservoirs, even though they have similar trophic status. This result is in agreement with that of a previous study where BCCs have been reported to be independent from trophic status but more dependent on the climate [7]. Several multi-lake studies have also shown variation in BCCs between different lakes, even those of similar trophic status [6,7,28].

The clusters including *Proteobacteria*, *Actinobacteria*, *Bacteroidetes*, and *Cyanobacteria* were found in all the three reservoirs, and they constituted the majority of the bacterial community in the analyzed reservoirs. This is consistent with the results found from similar reservoirs [9,29]. The phylogenetic grouping analysis suggests that water bodies with different eutrophic status have different bacteria community structures. In a trophic water body, the proportion of *Proteobacteria* and *Bacteroidetes* is higher compared with that in a less trophic water body. Hence, it is suggested that those two types of bacteria may have a positive correlation with the trophic level of freshwater bodies. In Chaohu Lake, both spatial and temporal variations in the richness and diversity of bacterioplankton communities have been observed, and the BCCs in 20 samples are substantially different at different seasons and locations. The seasonal difference accounts for most of the variation [8]. Thus, it appears that seasonal change is the most important factor affecting BCC in those three reservoirs, rather than trophic status. Shifts of BCC are, to a great extent, the consequence of fluctuations in environmental factors. Understanding temporal and spatial variability of bacterial communities requires frequent sampling from diverse locations. The present work only examines patterns of change in BCCs derived from monthly samples and at only one station in each reservoir. This prevents determination as to whether this is a multi-year effect with a cyclical pattern. Further long-term monitoring with multi-stations is needed to clarify the relationship between BCC and water environment. Nevertheless, it is clear that seasonal change is the dominant factors of BCC change in the Shenzhen freshwater reservoirs, which provides a foundation for microorganism control, and thus water quality control for those drinking source water bodies.

### 4.2. Effects of PCR-DGGE Techniques on Change Patterns of the BCC

The pattern of bands obtained by successful DGGE analysis represent the major constituents of the analyzed community. However, species that contribute less than 1% of the total population cannot be readily detected by this molecular approach [16,30]. In addition, since the DGGE technique focuses on the 16S-rDNA, which is more conserved than the intergenic transcribed spacer, it may lack the taxonomic resolution to distinguish between populations of closely related microorganisms. For instance, bands found at the same positions on the gels do not always represent the same taxon [31]. Therefore, differences and similarities in gel patterns may not completely correspond to the differences and similarities in nature. Moreover, it has been reported that BCC resulted from DGGE is influenced by DNA extraction methods after comparing four different DNA extraction methods [32]. Although the DGGE technique did not allow a complete characterization of the bacterioplankton community, the technique is capable of detecting differences in taxa composition among different samples [29,33], and can be used to assess the changes in BCC [30]; however, the relationship between DGGE bands and nucleotide sequences are difficult to establish. It is necessary to compare the DNA extraction methods and procedures, and then construct a 16S rDNA library for analyzing the characteristics of BCC bacterioplankton for actual bacterial diversity and phylogenetic analysis in the environment. In the meantime, we also acknowledge that our approach may introduce a bias to the DGGE analysis because not all the reservoirs have the same bacterial abundances and phylogenetic groups.

## 5. Conclusions 

We have successfully analyzed the BCC in three freshwater reservoirs, which are drinking water sources for the Shenzhen area. We found that seasonal climate change is the single most dominant effect of BCC. This provides a foundation for water quality control to focus on the seasons that exhibit high fluctuation.

## Figures and Tables

**Table 1:** Annual physico-chemical characteristics of the three study reservoirs.

Physico-Chemical Characteristics	Reservoirs		
	Shi-Yan	Xi-Keng	Luo-Tian
Surface area (km^2^)	6.33	3.87	3.28
Mean depth (m)	4.6	3.5	3.1
Total phosphorus (mg/L)	0.04	0.03	0.03
Total nitrogen (mg/L)	1.26	0.93	0.95
Chlorophyll a (μg/L)	27.01	13.39	39.52
Transparency (cm)	55.3	82.7	65.8
Dissolved organic carbon (DOC) (mg/L)	8.27	7.95	8.39
Permanganate Index (mg/L)	2.76	1.72	1.95
Temperature (°C)	25.48	25.24	25.95
Karlson TSI_M_ (modified tropic state index)	59.49	53.99	60.17

**Table 2:** The proportion of bacterioplankton community structure in the three reservoirs.

Bacteria	Reservoirs		
	Shi-Yan	Xi-Keng	Luo-Tian
*Proteobacteria*	40%	21.4%	21.4%
*Actinobacteria*	20%	14.3%	28.6%
*Acidobacteria*	/	7.1%	21.4%
*Bacteroidetes*	20%	14.3%	14.3%
*Cyanobacteria*	6.7%	21.4%	7.1%
*Planctomycetes*	/	/	7.1%
*Firmicutes*	13.3%	21.4%	/

## Abbreviations

The following abbreviations are used in this manuscript:

BCC	Bacterioplankton Community Composition’s
DGGE	Denaturing Gradient Gel Electrophoresis
TN	Total Nitrogen
TP	Total Phosphate
VBNC	Viable But Not Culturable
T-RFLP	Terminal Restriction Fragment Length Polymorphism
DOC	Dissolved Organic Carbon
UPGMA	Unweighted Pair-Group Method with Arithmetic
PCR	Polymerase Chain Reaction

## References

[1] Matveev V., Robson B.J. (2014). Aquatic food web structure and the flow of carbon. Freshw. Rev..

[2] Beisner B.E. (2001). Plankton community structure in fluctuating environments and the role of productivity. Oikos.

[3] Bianchi F., Acri F., Aubry F.B., Berton A., Boldrin A., Camatti E., Cassin D., Comaschi A. (2003). Can plankton communities be considered as bio-indicators of water quality in the Lagoon of Venice?. Mar. Pollut. Bull..

[4] Lai X.T., Zeng X.F., Fang S., Huang Y.L., Cao L.X., Zhou S.N. (2006). Denaturing gradient gel electrophoresis (DGGE) analysis of bacterial community composition in deep-sea sediments of the South China Sea. World J. Microb. Biot..

[5] Eiler A., Bertilsson S. (2007). Composition of freshwater bacterial communities associated with *Cyanobacterial* blooms in four Swedish lakes. Environ. Microbiol..

[6] Lindstrom E.S. (2000). Bacterioplankton community composition in five lakes differing in trophic status and humic content. Microb. Ecol..

[7] Yannarell A.C., Kent A.D., Lauster G.H., Kratz T.K., Triplett E.W. (2003). Temporal patterns in bacterial communities in three temperate lakes of different trophic status. Microb. Ecol..

[8] Wei C.L., Bao S.M., Zhu X.Y., Huang X.M. (2008). Spatio-temporal variations of the bacterioplankton community composition in Chaohu Lake, China. Prog. Nat. Sci..

[9] Lymperopoulou D.S., Kormas K.A., Karagouni A.D. (2012). Variability of prokaryotic community structure in a drinking water reservoir (Marathonas, Greece). Microb. Environ..

[10] Fisher M.M., Klug J.L., Lauster G., Newton M., Triplett E.W. (2000). Effects of resources and trophic interactions on freshwater bacterioplankton diversity. Microb. Ecol..

[11] Rösel S., Allgaier M., Grossart H.P. (2012). Long-term characterization of free-living and particle-associated bacterial communities in Lake Tiefwaren Reveals distinct seasonal patterns. Microb. Ecol..

[12] Newton R.J., Mcmahon K.D. (2011). Seasonal differences in bacterial community composition following nutrient additions in a eutrophic lake. Environ. Microbiol..

[13] Ramamurthy T., Ghosh A., Pazhani G.P., Shinoda S. (2014). Current perspectives on viable but non-culturable (VBNC) pathogenic bacteria. Front. Public Health.

[14] Schleifer K.H. (2004). Microbial diversity: Facts, problems and prospects. Syst. Appl. Microbiol..

[15] Lemke M.J., Leff L.G. (2006). Culturability of stream bacteria assessed at the assemblage and population levels. Microb. Ecol..

[16] Pires A.C., Cleary D.F., Almeida A., Cunha A., Dealtry S., Mendonça-Hagler L.C., Smalla K., Gomes N.C. (2012). Denaturing gradient gel electrophoresis and barcoded pyrosequencing reveal unprecedented archaeal diversity in mangrove sediment and rhizosphere samples. Appl. Environ. Microbiol..

[17] Ding T., Palmer M.W., Melcher U. (2013). Community terminal restriction fragment length polymorphisms reveal insights into the diversity and dynamics of leaf endophytic bacteria. BMC Microbiol..

[18] Osborn A.M., Moore E.R.B., Timmis K.N. (2000). An evaluation of terminal-restriction fragment length polymorphism (T-RFLP) analysis for the study of microbial community structure and dynamics. Environ. Microbiol..

[19] Yan Q.Y., Yu Y.H., Feng W.S., Yu Z.G., Chen H.T. (2008). Plankton community composition in the Three Gorges Reservoir Region revealed by PCR-DGGE and its relationships with environmental factors. J. Environ. Sci. Chin..

[20] Dar S.A., Kuenen J.G., Muyzer G. (2005). Nested PCR-denaturing gradient gel electrophoresis approach to determine the diversity of sulfate-reducing bacteria in complex microbial communities. Appl. Environ. Microb..

[21] Carlson R.E. (1977). A trophic state index for lakes. Limnol. Oceanogr..

[22] Gilcreas F.W. (1966). Standard methods for the examination of water and wastewater. Am. J. Public Health Nations Health.

[23] Muyzer G., De Waal E.C., Uitterlinden A.G. (1993). Profiling of complex microbial populations by denaturing gradient gel electrophoresis analysis of polymerase chain reaction-amplified genes coding for 16S rRNA. Appl. Environ. Microb..

[24] Juck D., Charles T., Whyte L., Greer C. (2000). Polyphasic microbial community analysis of petroleum hydrocarbon-contaminated soils from two northern Canadian communities. FEMS Microbiol. Ecol..

[25] Rölleke S., Muyzer G., Wawer C., Wanner G., Lubitz W. (1996). Identification of bacteria in a biodegraded wall painting by denaturing gradient gel electrophoresis of PCR-amplified gene fragments coding for 16S rRNA. Appl. Environ. Microb..

[26] McCaig A.E., Glover L.A., Prosser J.I. (2001). Numerical analysis of grassland bacterial community structure under different land management regimens by using 16S ribosomal DNA sequence data and denaturing gradient gel electrophoresis banding patterns. Appl. Environ. Microb..

[27] Hill T.C.J., Walsh K.A., Harris J.A., Moffett B.F. (2003). Using ecological diversity measures with bacterial communities. FEMS Microbiol. Ecol..

[28] Newton R.J. (2008). Cosmopolitan Freshwater Bacterial Dynamics in Lakes across Time and Space.

[29] Luo W., Chen H., Lei A., Lu J., Hu Z. (2014). Estimating *Cyanobacteria* community dynamics and its relationship with environmental factors. Int. J. Environ. Res. Public Health.

[30] Duarte S., Cassio F., Pascoal C. (2012). Denaturing gradient gel electrophoresis (DGGE) in microbial ecology—Insights from freshwaters. Gel Electrophor. Princ. Basics.

[31] Tatiana V., Edward T., Gerard M., Valérie M., Gisèle L., Annabel R., Guy S. (1997). Evaluation of denaturing gradient gel electrophoresis in the detection of 16S rDNA sequence variation in rhizobia and methanotrophs. FEMS Microbiol. Ecol..

[32] Hang M., Xiao-Yu X., Zhen-Mei L., He L. (2006). Comparison of DNA extraction methods for PCR-DGGE analysis of the soil bacterial community. Chin. J. Agric. Biotechnol..

[33] Tian J., Lu J., Zhang Y., Li J.C., Sun L.C., Hu Z.L. (2014). Microbial community structures and dynamics in the O3/BAC drinking water treatment process. Int. J. Environ. Res. Public Health.

